# Benchmarking Attention-Based Interpretability of Deep Learning in Multivariate Time Series Predictions

**DOI:** 10.3390/e23020143

**Published:** 2021-01-25

**Authors:** Domjan Barić, Petar Fumić, Davor Horvatić, Tomislav Lipic

**Affiliations:** 1 Department of Physics, Faculty of Science, University of Zagreb, Bijenička cesta 32, 10000 Zagreb, Croatia; domjanbaric@gmail.com (D.B.); pfumic@phy.hr (P.F.); 2 Division of Electronics, Ruđer Bošković Institute, Bijenička cesta 54, 10000 Zagreb, Croatia

**Keywords:** multivariate time series, attention mechanism, interpretability, synthetically designed datasets

## Abstract

The adaptation of deep learning models within safety-critical systems cannot rely only on good prediction performance but needs to provide interpretable and robust explanations for their decisions. When modeling complex sequences, attention mechanisms are regarded as the established approach to support deep neural networks with intrinsic interpretability. This paper focuses on the emerging trend of specifically designing diagnostic datasets for understanding the inner workings of attention mechanism based deep learning models for multivariate forecasting tasks. We design a novel benchmark of synthetically designed datasets with the transparent underlying generating process of multiple time series interactions with increasing complexity. The benchmark enables empirical evaluation of the performance of attention based deep neural networks in three different aspects: (i) prediction performance score, (ii) interpretability correctness, (iii) sensitivity analysis. Our analysis shows that although most models have satisfying and stable prediction performance results, they often fail to give correct interpretability. The only model with both a satisfying performance score and correct interpretability is IMV-LSTM, capturing both autocorrelations and crosscorrelations between multiple time series. Interestingly, while evaluating IMV-LSTM on simulated data from statistical and mechanistic models, the correctness of interpretability increases with more complex datasets.

## 1. Introduction

Applying deep learning (DL) models to multivariate time series data [1] has recently gained growing popularity in a variety of critical application domains such as climate, environment, healthcare [2], finance, as well as other social good domains [3] or Internet of Things driven critical infrastructures [4]. However, the adaptation of deep learning methodology within such safety-critical application scenarios and systems cannot rely only on prediction performance but has to provide human understandable, interpretable, and robust explanations for their decisions. In general, explainable artificial intelligence considers different methodologies, techniques, and tools for achieving explainability for different target users (e.g., machine learning experts, domain experts or general end users) [5] in various DL models by either directly using intrinsically interpretable (self-explainable) models [6] or by relying on post hoc (usually model agnostic) interpretability techniques such as popular Shapley additive explanations (SHAP) [7]. In her perspective work [6], Cynthia Rudin argues that we should focus on developing and using models that are inherently interpretable, as well as that we should be very careful when applying post hoc explanation techniques in high-stakes applications involving critical decision making. In addition, DL methodologies also have to incorporate human domain knowledge during training and learn and reason about human high-level cognitive concepts.

As a recently emerging trend of evaluating DL methodologies from a human high-level cognition perspective, different real-world and synthetically designed datasets were proposed and utilized defining and evaluating specific image, video, and text human high-level understanding tasks. These include now a long list of large-scale image fine art collection Wikiart for high-level subjective aspects of human perception (i.e., aesthetics, visual sentiment, and memorability) [8], Omniglot dataset for evaluating human-like concept learning [9], e-SNLI dataset providing human-annotated natural language explanations of the entailment relations [9], CLEVR dataset for question-answering based visual reasoning tasks [10], CATER dataset for compositional actions and temporal reasoning or even specifically constructed datasets for evaluating abstract reasoning with visual IQ tests based on Raven’s Progressive Matrices [11] or mechanics based on update rules of two-dimensional cellular automaton Conway’s Game of Life [12,13].

Most of these efforts were focused on neural architectures for a common DL application based on unstructured data modalities such as image, video, and text. Although, in recent years, time series forecasting with deep learning has been intensively studied [1], novel research trend for eventuating interpretability of deep learning models within time series prediction tasks has just started to emerge [14,15,16,17]. A recent survey by Lim and Zohran outlines the state-of-the-art techniques available for common forecasting problems based on DL architectures [1]. Recently, Ismail and co-authors introduced the first benchmark that systematically evaluates different saliency methods across multiple DL models for multivariate time series setting [16]. Furthermore, another study proposed a Performance-Explainability Framework to benchmark existing machine learning methods and then applied it to current state-of-the-art models for multivariate time series classification setting [18]. However, to best of our knoweldge currenlty there is no benchamark that allows interpretability analysis for multivariatie time series forecasting setting.

One common time series prediction task is (multivariate) forecasting, where a model given the historical values of one (or more) time series as input tries to predict the future values of one (or more) time series. In a multivariate setting, when more than one time series is used as input or output, forecasting output can be future values of all input variables (time series) or future output of just one independent input variable, which has some relationship with other input variables. Deep learning models based on encoder-decoder architectures are currently considered state-of-the-art for this task and for modeling complex sequential data, where different encoders such as convolution neural networks (CNN) with dilated convolution, recurrent neural networks (RNN), and attention mechanisms can be used to incorporating temporal information.

Attention mechanisms are also regarded as the established approach to support deep neural networks with intrinsic interpretability when modeling complex sequences. On the other hand, post-hoc interpretability methods typically disregard sequential dependencies between inputs and are not usually suited for application to time series data with complex causal associations. The mechanism is embedded into a model and learns the weighted average of hidden states from different time steps in a long sequence, allowing the model to focus on significant time steps in the past directly. One can interpret these attention weights as the strength of the causal relationship between different data points. A recent work on interpretability of attention distributions shows that the attention distributions could be utilized in specifically accustomed DL models to provide *faithful* and *plausible* explanations for models’ predictions [19]. If higher attention weights imply a more significant impact on the model’s predictions, then we consider attention distributions as faithful explanations, while we can also consider them as plausible explanations if they provide human-understandable justification for the model’s predictions [19]. In this paper, we set to benchmark attention-based models for multivariate time series prediction task. We investigate the predictive power of these models and interpretability correctness.

Equal weight is put on understanding if the interpretability of models is correct and how confident models are regarding interpretability. Using a custom time series, we can safely evaluate models’ interpretability because we know the underlying process for generating the data in detail. This is why we create new datasets and do not use one of the standard benchmarks, such as CauseMe [20,21,22] and M4 [23]. For the CauseMe dataset, one can know the type of underlying process, i.e., is time series generated by linear, logistic, or some other autoregressive process, but cannot determine if interpretability given by the model is correct (one will not be able to score it more quantitatively than the correct or wrong nature of the process). Furthermore, most of the time series in the CauseMe dataset only have autoregressive processes. They lack interactions between multiple time series, which is essential to our evaluation, and we want to determine if models can catch these interactions. M4 dataset consists of real-world data where there is no understanding of the underlying process. Data is transformed and masked so that it is impossible to know the origin of it. Furthermore, the M4 dataset has only a singular time series per example. Thus, by knowing the underlying process entirely and changing the parameters of processes that generate the data, we can better explore the domain of model performances and understand where they perform best and where they break.

This paper focuses on the emerging trend of specifically designing diagnostic datasets for understanding the inner workings of attention mechanism based DL models for multivariate forecasting tasks. Creating new synthetic datasets allowed us to choose interactions between time series. By knowing the underlying mechanics of the dataset, we were able to validate models’ interpretability, i.e., how models perform when we have variable complex data interactions. To the best of our knowledge, we are the first to create transparent datasets for multivariate-time series forecasting tasks and utilize these for prediction, stability, interpretability performance evaluation of neural architectures with inherent interpretability (by incorporating attention mechanism). Few recent studies aiming to interpret deep neural networks applied to time series data in multivariate setting does not cover interactions between time series (such as [18]) or are focused on evaluation on post hoc explainability techniques (such as [16]).

The main contributions of this work are:

- We design a novel benchmark of synthetically designed datasets with the transparent underlying generating process of multiple time series interactions with increasing complexity for understanding the inner workings of attention mechanism based deep learning models for multivariate forecasting tasks.
- Using the designed benchmark, we conduct a comprehensive analysis of the performance of existing attention based deep neural networks in three different aspects: prediction performance score, interpretability correctness, sensitivity analysis.
- We demonstrate that although most models have satisfying and stable prediction performance results, they often fail to give correct interpretability and that intrinsic interpretability increases with the complexity of interactions between multiple time series.

The rest of the paper is structured as follows: Section 2 contain an overview of the deep learning models for multivariate time series data that will be evaluated, in Section 3, we present details of experimental and evaluation framework/setup, Section 4 contains experimental results, and in Section 5 conclusions and outlook are presented.

## 2. Deep Learning Models for Multivariate Time Series Data

This paper aims to understand and benchmark how different DL models with inherent explainability perform, both in prediction performance (for example, accuracy of prediction) and quality of given interpretability, i.e., do models find correct causality between different time series. We focus on existing attention-based models because one can interpret attention coefficients as the model’s feature’s importance. Four models that will be analysed are:

- seq2graph [24]
- Interpretable Multi-Variable Long short-term memory (IMV-LSTM) [25]
- Temporal Causal Discovery Framework (TCDF) [26]
- Dual Stage attention (DA-RNN) [27]

### 2.1. seq2graph

The idea behind the seq2graph model is to use gated recurrent units (GRU) to create a directed weighted graph from multivariate time series. The architecture consists of four different parts: Encoder, Dual-purpose RNN, Transformation Layer, and Decoder.

The Encoder is a bidirectional GRU unit. Outputs *h* from the Encoder are concatenated with input into the model and then sent to the Dual-Purpose RNN. The architecture of the dual-purpose layer is the same as the Decoder layer. Both calculate attention scores from layer input. This input is multiplied with attention scores and then fed through the GRU unit, with tanh activation applied in the end. Attention coefficients in Dual-purpose RNN are called α coefficients, and they model autocorrelation (Equation (5) in [24]). For each time series in the dataset at time *t*, and the window size *w*, of RNN layer in the neural network, we have *N*α coefficients, where *N* is the number of time-series in the dataset, and each α coefficients has *w* values. As these are attention coefficients, they sum to 1. Attention coefficients in the Decoder layer are called β coefficients (Equation (9) in [24]), and they model crosscorrelation. Similar to α coefficients, we have *N*β coefficients, and β coefficients also sum to 1. Dual-purpose RNN layer produces sequence $v^{d}=v_{1}^{d},v_{2}^{d},\ldots ,v_{m}^{d}$ for *d*-th time series. Dual-Purpose RNN and Encoder work on each time series separately. The role of the transformation layer is to join outputs from different time series to one feature vector. The output from every Dual-purpose RNN is concatenated, so that sequence $v^{d}$ becomes a feature vector, and we introduce time dimension as the ordering of time series, i.e., we create new vector $W=v^{1},v^{2},\ldots ,v^{d}$. This vector is used as input to the Encoder layer, which produces a prediction for each time series. seq2graph model is the only one that allows us to train the model for each time series simultaneously. Other models require that we train a different model for each time series. It is important to note that we added a linear layer at the end of the seq2graph model to predict outside default range [−1,1].

### 2.2. Interpretable Multi-Variable Long Short-Term Memory

Interpretable Multi-Variable Long short-term memory (IMV-LSTM) model introduces a novel recurrent unit. The idea of IMV-LSTM is to make use of the hidden state matrix and develop an associated update scheme, such that each element (e.g., row) of the hidden matrix encapsulates information exclusively from a specific variable of the input. The hidden state vector is replaced with a hidden state matrix, where each *n*-th matrix row corresponds to *n*-th input variable. Cell input activation vector is replaced with the hidden state update, which is defined as: $\tilde{j_{t}}=\tanh\left(W_{j}⊗{\tilde{h}}_{t-1}+U_{j}⊗x_{t}+b_{j}\right)$ (Equation (1) in [25]), where ${\tilde{h}}_{t-1}$ is hidden state matrix and $x_{t}$ is input vector. Parameters $W_{j}$ and $U_{j}$ represent layer weights, while $b_{j}$ represents bias vector. There are two different instances of IMV-LSTM: IMV-LSTM-Full, which behaves in the same way as standard LSTM but enjoys interpretability (achieved by changing the hidden update vector by ${\tilde{j}}_{t}$, and IMV-LSTM-Tensor, where gates and memory cells are matrices as well. In IMV-LSTM gates only scale hidden state and memory cell, so variable-wise organizations in the data hidden state are preserved. To further deepen intuition with IMV-LSTM, one can think about IMV-LSTM-Tensor as a set of parallel LSTMs, where each processes one variable series and then merges via the mixture. The hidden states, specific to each variable, are derived as a mixture of auto and cross-correlation mechanisms. Same as seq2graph, IMV-LSTM model has α and β coefficients that model autocorrelation and crosscorrelation respectively. The hidden state matrix **$\tilde{h}$** is used as input to calculate attention coefficients α. Product of α and hidden state matrix **$\tilde{h}$** is concatenated with **$\tilde{h}$**, i.e., $\left[\alpha *\tilde{h},\tilde{h}\right]$, and used as input to attention mechanism to calculate β coefficients.

### 2.3. Temporal Causal Discovery Framework

Temporal Causal Discovery Framework (TCDF) consists of *N* independent attention-based Convolutional neural networks (CNNs), all with the same architecture but with a different target time series, i.e., different models for predicting each time series. TCDF is based on generic Temporal Convolutional Network (TCN) architecture. TCDF uses multiple convolutional layers with dilatation to increase the receptive field, and to deal with the accuracy degradation problem introduced by multiple layers, TCDF model uses residual connections. Target time series is also used as input to model autocorrelation. To model cross-correlation, TCDF extends TCN architecture by introducing one-dimensional depthwise separable convolution. Channels are kept separate by applying a different kernel to each input channel, followed by a 1×1 pointwise convolution. Interpretability is gained by introducing an attention mechanism. TCDF implements attention as a trainable 1×N-dimensional vector that is element-wise multiplied with the *N* input time series. Authors of TCDF argue that one would prefer hard attention over soft attention. To bridge this gap between soft attention used in the model and hard attention, which is preferred, TCDF applies a semi-binarization function.

### 2.4. Dual Stage Attention

Dual stage attention (DA-RNN) architecture consists of four different parts: Input attention, Encoder, Temporal Attention, and Decoder. The input attention mechanism computes the attention weights for multiple driving series. Attention weights are multiplied by input and then fed to the LSTM encoder unit. The temporal attention system computes the attention weights based on the previous decoder’s hidden state. As Decoder input, the sum of the attention weighted encoder hidden states is used. The output of the last decoder LSTM unit is the predicted result. It is important to note that all other neural networks that we analyse try to model autocorrelation first and crosscorrelation after, while DA-RNN tries to choose driving time series, i.e., models crosscorrelation, as the first step, and then tries to find temporal causation, i.e., models autocorrelation.

### 2.5. Overview of Analyzed Deep Learning Models

**Figure 1:**
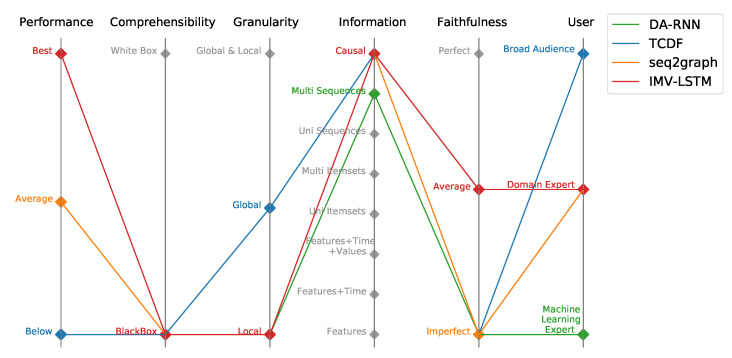
Parallel coordinates plot of evaluated models within Performance-Explainability Framework.

The overview and classification of evaluated models is presented in Figure 1 according to the recently introduced Performance-Explainability Framework, which incorporates six major evaluation components (performance, comprehensibility, granularity, information, faithfulness, user). The performance component in the framework assesses the performance of DL models and corresponds to the relative performance of a model on a particular application and evaluation setting compared to the state-of-the-art model. Comprehensibility corresponds to the ability of the user to understand how the model works and produces certain predictions. The granularity component tells us on which level the model gives explainability, i.e., if the explanation is given to a single prediction (Local) or given for overall model behavior (Global). The information component describes the type of information that is given by the model to the user. The faithfulness component tells us how much an end-user can trust the explanations of model predictions. The user category indicates the audience to whom the explanations are accessible, i.e., how much prior knowledge is needed to understand model explanations.

## 3. Experimental and Evaluation Framework/Setup

### 3.1. Synthetic Time Series Datasets

To analyze model performance in prediction accuracy and interpretability, we created set of synthetic data from models presented in Table 1.

**Figure 2:**
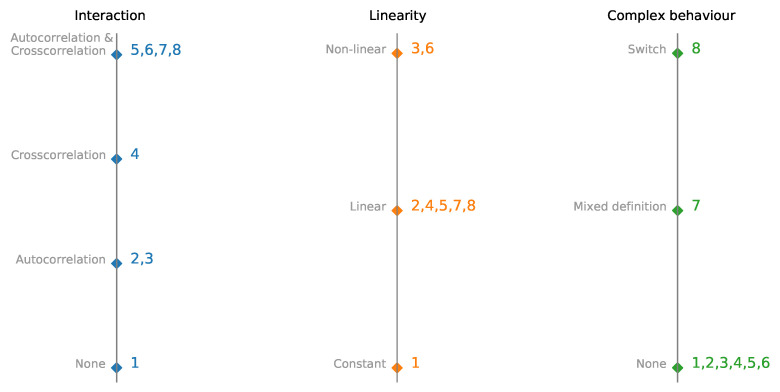
Parallel coordinates plot of multivariate time-series synthetic datasets with embedded interactions, linearity, and complexity. Datesets are presented in detail in Table 1.

Each dataset consists of *N* time series, and with each successive dataset, we increase the model’s complexity. We start with a set of N constant time series (dataset 1). For datasets 2 and 3, time series are autoregressive and nonlinear autoregressive, and there is no interaction between time series. Dataset 4 consists of two interdependent time series without autoregression. The next two sets consist of one (nonlinear) autoregressive time series, and all other time series are calculated from autoregressive (dataset 5 and 6). Datasets 2 and 5 and datasets 3 and 6 have similar dynamics, and nonlinear activation is added to see the impact of nonlinearity on model performance and interpretability. Dataset 7 is a custom vector autoregression model. The switching time series are used for dataset 8. Depending on the first time series’s value, the next step is generated by a different set of rules. Differences between datasets 1–8 are plotted in Figure 2 to visualize interactions, linearity, and complexity better.

Additionally we created two datasets from statistical and mechanistic models. Logistic map inspired model and Ising model on first-order 2D square lattice was used to generate dataset 9 and 10. Logistic map is defined as $x_{n+1}=rx_{n}\left(1-x_{n}\right)$, so we constructed a model for multiple time-series in dataset 9 using *r* coefficient equal to:

- 1.5—time-series quickly converge to (1.5–1)/1.5
- 2.5—time-series converge to (2.5–1)/2.5 but will fluctuate a bit before
- 3.2—time-series oscillate between two values
- 3.55—time-series oscillate between more than 4 values
- 3.56996—time-series enter chaotic domain

A logistic map is of particular interest because we can have chaotic behavior generated by a simple rule. For both Ising and logistic map inpired model, there is no need to incorporate noise into data. All examples in other tests need noise, or they converge to a single value. Once we enter a single value space, causality becomes trivial.

For Ising model [28,29], first-order means that spins only interact with their closest neighbors. We use 2D squared lattice, i.e., each spin has four neighbors, and the dimension of the lattice is 10×10. 2D squared lattice is the simplest lattice in which we can observe phase transition. We analyze lattice at a temperature equal to 2, $T_{c}$, and 2.75. Phase transition should be observed at temperature $T_{c}=2.269$ in dimensionless units.

Datasets 1–8, and 10 were generated in the same manner. For each time series in the dataset, we sample 10 points from the normal distribution $N(\mu =0,\;\sigma^{2}=1)$, and then these points are used as initial points for a time series. After initializing values are set, we generate values at each time step given by a model in Table 1. Values are generated sequentially through time. For datasets 1–8, we add Gaussian noise $\epsilon_{t}$ with probability *f* at each time step. Value of noise is sampled from a normal distribution with mean equal to $\mu_{noise}$ (for all of our experiments, the value of this parameter is equal to 0) and standard deviation equal to $\sigma_{noise}^{2}$:

$$\epsilon_{t}=\left\{\begin{array}{cc} N(\mu =\mu_{noise},\;\sigma^{2}=\sigma_{noise}^{2}), & p=f,\\ 0, & p=1-f. \end{array}\right.$$

To generate dataset 9, we use first-order 2D squared Ising lattice where spins only interact with their closest neighbors, i.e., each spin has four neighbors. The dimension of the lattice is 10 × 10. 2D squared lattice is the simplest lattice in which one can observe the phase transition at the temperature of $T_{c}=2/\ln\left(1+\sqrt{2}\right)=2.269$ (in dimensionless units). For state at t=0 we sampled 100 values of either: state up (value equal to 1) with probability 0.5 or state down (value equal to 0) with probability 0.5. All other points are generated using Metropolis algorithm. For each time-series in datasets 1–10 we generated 20,000 points and discarded first 1000. Code used for the generation of all presented datasets is available on the GitHub repository (https://github.com/hc-xai/mts-interpretability-benchmark).

### 3.2. Quantitative Evaluation—Prediction Performance

Noise parameters used in datasets 1–8 are:

- $\mu_{noise}=0$
- $\sigma_{noise}^{2}=0.1$
- f=0.3
- *N* = 5 (except for dataset 4 *N* = 2, and dataset 7, 8 *N* = 4)

Since there is no need to incorporate noise into data for Ising model (dataset 9), and logistic map inspired model (dataset 10), we used $T_{c}=2/\ln\left(1+\sqrt{2}\right)=2.269$ (phase transition), and r=3.56996 (chaotic domain) respectively. All interactions in data had lag lower than 10. We also used a window of 10 while training and testing the model. We used the window the same as maximal lag because we do not test how models perform if some data is missing, i.e., we evaluate models when all needed information is available. Every time series was generated with a length of 20,000. The last 2000 points were used as a test set. Mean squared error (MSE) was used as an error for evaluation.

In every experiment, MSE was calculated for each single time series. These errors were averaged and reported as experiment error. Multiple experiments were run for each model:

- IMV-LSTM—3 experiments
- seq2graph—5 experiments
- TCDF—10 experiments
- DA-RNN—3 experiments

The number of experiments per model varies because of the time needed to train and evaluate the model. From error on a single experiment, we calculate average error and standard deviation of error. This is done on train data and test data and reported as a model error.

### 3.3. Qualitative Evaluation—Interpretability

To analyze if models learn correct causality, we take a closer look at the models’ interpretability. Datasets used to evaluate interpretability are 2, 4, 8, 9, and 10. These data are used to distinguish how models learn and discover autocorrelation or crosscorrelation (or any higher-order interactions). Hyperparameters are the same as those used in quantitative analysis. Causality is taken across multiple experiments. We report both the average value of causality and standard deviation. We want to understand what causality the model has learned and how it corresponds with true causality with the average value. Using standard deviation, we can understand how stable this causality is from experiment to experiment. We look both at the temporal causality and cross time-series causality.

### 3.4. Sensitivity Analysis—Dependence on Hyperparameters

To evaluate how models perform for different values of parameters used in data generation ($\mu_{noise}$, $\sigma_{noise}^{2}$, *f*, *N*), we perform what we call sensitivity analysis. We vary one parameter while keeping other parameters fixed. Values of these other parameters are the same as those we used in quantitative analysis Section 3.2. This analysis is done for three parameters: $\sigma_{noise}^{2}$, *f*, and *N*. Varying $\mu_{noise}$ was not considered because time-series diverge more often than not if we vary $\mu_{noise}$ significantly while keeping other parameters fixed.

Every sensitivity analysis test was run on two different datasets: one which should be considered easier and one which should be considered more challenging. For example, dataset 7 is the more challenging dataset for noise frequency *f* and amount of noise $\sigma_{noise}^{2}$ and for the number of time series *N* more challenging dataset is dataset 5. Parameters range is specified as follows:

- Noise frequency: *f*—from 0 to 1 with step 0.05
- Noise amount: $\sigma_{noise}^{2}$—values are: 0.01, 0.05, 0.1, 0.2, 0.5, 1, 2, 3, and 5
- Number of time series: *N*—from 3 to 20 with step 2

The experiment was run three times for every model except for seq2graph, where we ran the experiment 5 times. Same as in quantitative analysis, time series was generated with a length of 20,000, with the last 2000 points taken as the test set. MSE was used as an error for evaluation, and it is reported in the same manner.

## 4. Experimental Results

In this section, we present the results of our experiments. First, we show the performance of models based on their predictive power. Next, we discuss interpretability correctness and stability given by models. After that, we test how models perform when we change the parameters of data generation. Finally, we look at IMV-LSTM performance on data from statistical and mechanistic models (dataset 9 and dataset 10). The code used for experiments is written in the Python programming language and all neural network models are implemented in PyTorch. IMV-LSTM, TCDF, and DA-RNN are initiated and trained with default parameters available on their GitHub repositories. The authors implemented the seq2graph model, as the code was not available with the original paper. For training of seq2graph, we use Adam optimizer, with a learning rate of 0.01/3, batch size of 128, and 5 epochs. The training and prediction for all models were performed on Intel(R) Xeon(R) Gold 6142 CPU, with 252 GB of RAM, and an NVIDIA GeForce RTX 2080 Ti GPU.

### 4.1. Quantitative Analysis

In Table 2, we can see the average experiment MSE for each model. The IMV-LSTM model is the best performing one across most datasets, followed by DA-RNN and seq2graph models. Models follow similar error patterns across datasets. It is interesting to see that the TCDF model has slight deviations from this error pattern, both in performance (measured by average MSE) and stability (measured by the standard deviation of MSE). TCDF is the only model that uses convolution layers, while all other models use sequential layers. It is also interesting to see that IMV-LSTM is the worst performing model on dataset 1, which should be the easiest. Furthermore, for IMV-LSTM, the average MSE on this dataset is lower than the average MSE on dataset 8. In Table 2, we also added the performance of Exponential Smoothing RNN (ES-RNN) model [30] on Datasets 1–8 for comparison. ES-RNN model is the winner of the M4 competition and it uses exponential smoothing to capture non-stationary trends and learns additional effects with the RNN. In general, hybrid models utilize deep neural networks in two manners: to encode time-varying parameters for non-probabilistic parametric models, and to produce parameters of distributions used by probabilistic models. We do not further analyse ES-RNN because it does not provide interpretability to user. Furthermore, ES-RNN has univariate input, not multivariate, i.e., it takes single time series as input.

**Figure 3:**
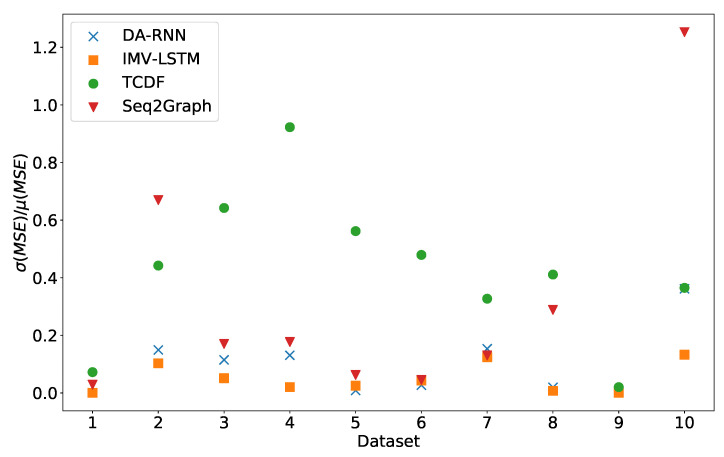
Stability of prediction performance by dataset. On y-axis we plot standard deviation of MSE divided by mean value of MSE. The x-axis corresponds to the index of dataset. As we can see TCDF model is the model with most unstable performance across most datasets, with seq2graph having worse performance on datasets 2 and 10. IMV-LSTM is the model with most stable performance.

In Figure 3, we can see the standard deviation of experiment error divided by average experiment error. We can see that the IMV-LSTM model is the best performing model with the lowest variance. TCDF is a model with the highest variance across all datasets. It is interesting to see that the constant time series has a low standard deviation, which means that models constantly perform worse than other on datasets (excluding dataset 7 and 8).

### 4.2. Qualitative Analysis

Interpretability given by seq2graph, TCDF, DA-RNN, and LSTM models will be discussed in this section.

#### 4.2.1. seq2graph

**Figure 4:**
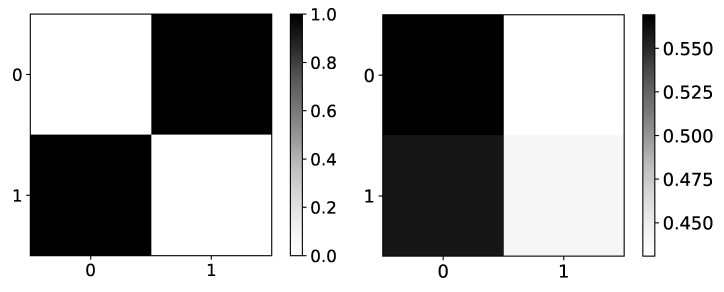
Ground truth (left panel) and mean β values (right panel) from seq2graphs model for dataset 4. The y-axis corresponds to the target series index, and the x-axis corresponds to the index of series whose impact on selected target series we plot. This dataset consists of two time-series that are generated from each other without autoregression included. This is the reason we expect high values on antidiagonal. β coefficients of seq2graph differ from what we expect, and they are biased towards time-series with index 0.

In the left panel in Figure 4, we show expected values of β coefficients for dataset 4. Rows are indices of the target series that we are predicting, while columns represent the impact of series *i* on the target series (as in all figures that show β coefficients). As dataset 4 consists of two time-series, where first is generated by second and vice versa, we expected to see high values on antidiagonal and low values on the main diagonal. In the right panel in Figure 4, we can see mean β values given by seq2graph on dataset 4. Seq2graph is biased towards the first time series in the dataset, which is seen across most datasets.

We believe this problem occurs because of artificial transformation from feature vector to time series vector before Decoder RNN part of the model. The feature vector from each time series is concatenated into one sequence, and the order of the time series is introduced as a time dimension.

**Figure 5:**
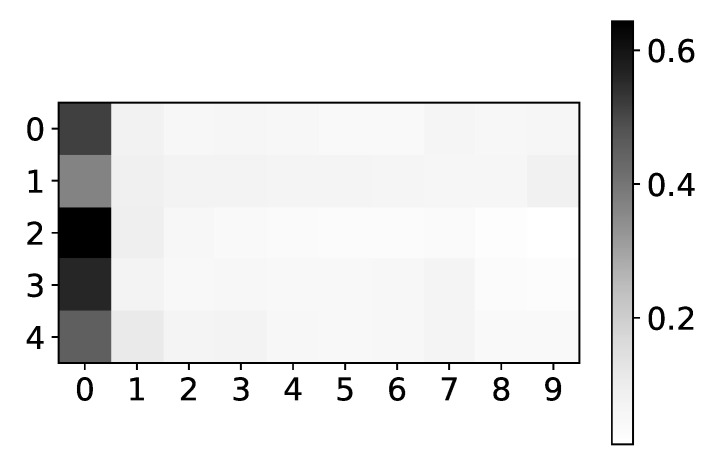
The mean values of α coefficients of seq2graph model for dataset 2. The y-axis corresponds to the index of target series and x-axis corresponds to the time lag whose impact on selected target series we plot. We can see seq2graph bias towards beginning of the window.

In Figure 5 we plot mean α coefficients for dataset 2. Same as the plot for β coefficients, the y-axis shows the target series index, while the x-axis shows the time lag. The color shows the value of α. We would expect to see high values for lags 3 and 7, for all time-series, and low values for all other lags. As we can see, the seq2graph model is biased towards the beginning of the window. Bias towards the beginning or towards the end of the window is seen across all datasets. We suspect that this bias comes from using LSTM cells.

Furthermore, the interpretability we get from the seq2graph model does not have high confidence. To show this, we plot mean β coefficients for dataset 2. For this dataset, we used N=5. It consists of only autoregressive time-series, i.e., there is no interaction between time-series, so we expect to have high values on the main diagonal, and all other values should be close to zero.

**Figure 6:**
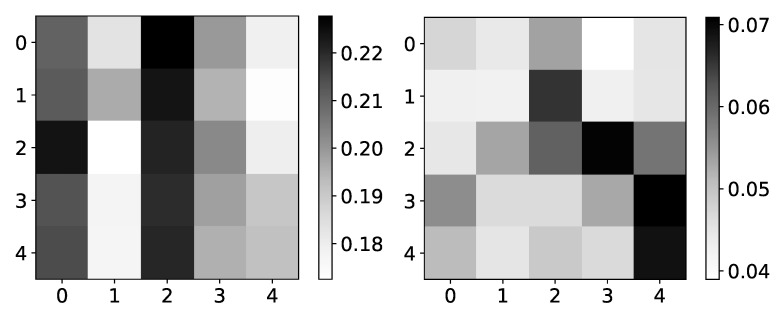
Mean (left) and standard deviation (right) of β coefficients from seq2graphs model for dataset 2. The y-axis corresponds to the target series index, and the x-axis corresponds to the index of series whose impact on selected target series we plot. In the left panel, we plot the mean values of β coefficients of seq2graph model for dataset 2. Similar to the analysis on Figure 4, we can see that seq2graph is biased to one time series. In this case, it is time series with index 2 and a lesser extent time series with index 0 (i.e., beginning of the window). We plot the standard deviation of β coefficients in the right panel, averaged across all experiments. High standard deviation with almost uniform mean values suggests low confidence in given interpretability.

The left panel in Figure 6 shows mean β values for dataset 2 across three different experiments. The maximum value is 0.22, and the minimum value is 0.18. In the right panel in Figure 6, we can see the standard deviation of the same dataset’s β values. The minimum value is 0.04. With uniform mean β values, high standard deviation suggests that the seq2graph model’s interpretability is highly uncertain, even though the model has satisfying quantitative analysis scores. It is important to notice that if there were no interpretability, i.e., all attention values are equal, we would see a beta value of 0.2 (as they should sum to 1 for single target time series). When you take in consideration that mean β values are in range [0.18,0.22] with standard deviation of ∼0.05, statistically, β values are not different from value of 0.2.

#### 4.2.2. TCDF

**Figure 7:**
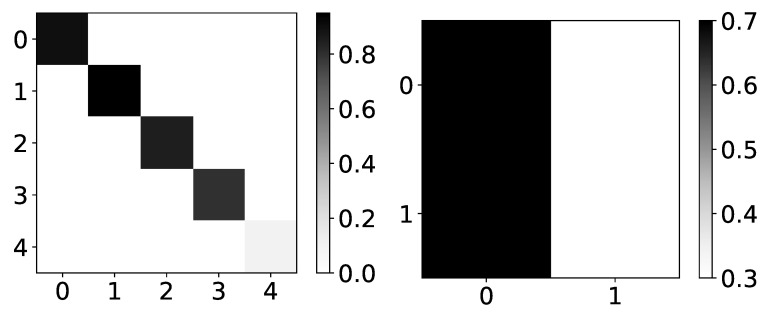
Percentage of TCDF retrieved causality association within series in dataset 2 and 4. On the y-axis, we plot the target time series. The x-axis corresponds to the index of the series whose impact on the selected target series we count. Values in a single row do not need to sum to 1 for TCDF because there can be a case where TCDF did not find any causality for the targeted time series. These are not attention coefficients. In the left panel, we plot the percentage of experiments that TCDF did find causality for dataset 2. We can see that interpretability is correct for this dataset. However, for the last time series, it found causality in a small percentage of experiments. It is crucial to notice that TCDF never found incorrect causality in this dataset. In the right panel, we plot the percentage of experiments that TCDF did find causality for dataset 4. For this dataset, TCDF always says that a single time series is generating both of them. In the majority of experiments, it was time series with index 0.

As mentioned in Section 2.3, TCDF applies semi-binarization to attention outputs to achieve hard attention. We look at these “hard” outputs in the analysis. In the left panel in Figure 7 we can see the heatmap of percentage of retrieved causal relationships given by the TCDF model on dataset 2. TCDF gives correct interpretability, but it does not find it in every experiment. This is especially seen on the last time series in dataset 2, where only in 10% of cases model found any causality. However, when TDCF does recognize causality, it is correct. The potential reason for this could be that it is necessary to decide the threshold of importance for the model, which could vary from dataset to dataset highly. Furthermore, it is a bit impractical to have to choose this threshold. It either means that one needs to run the model multiple times, which introduces human bias and is prone to overfitting, or one has to choose it beforehand, which introduces high uncertainty to interpretability. In the right panel in Figure 7 we plot heatmap of percentage of retrieved causal relationships for dataset 4. Here we find interesting behavior of TCDF. On dataset 4, if TCDF finds causality, it says that single series is generating both series in the dataset. In 70% of experiments, it is time series with index 0 and in 30% experiments, it is a time series with index 1.

**Figure 8:**
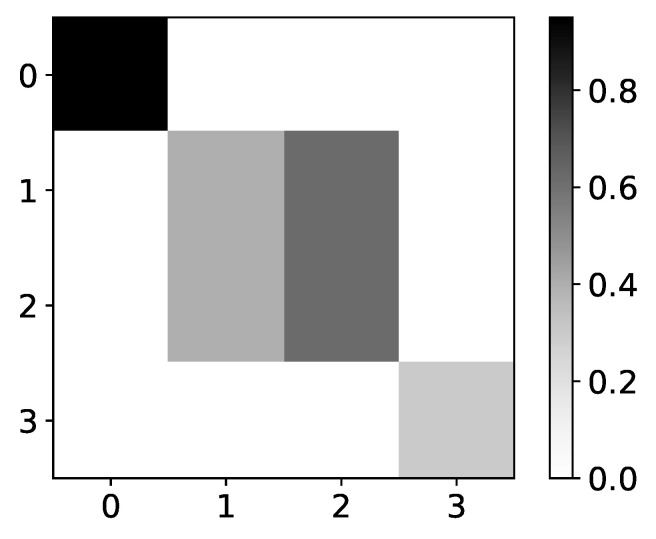
Percentage of TCDF retrieved causality association within series in dataset 8. On y-axis we plot target time series. The x-axis corresponds to the index of series whose impact on selected target series we count. Values in single row do not need to sum to 1 for TCDF, because there can be an experiment where TCDF did not find any causality for targeted time series. In this figure we plot interpretability given by TCDF on dataset 8. We can see that TCDF gives correct interpretability for time series with index 0 and 3, whose behaviour is autoregressive. For time series with index 1 and 2, this model gives wrong interpretability. Furthermore, even though model gives correct interpretability for time series with index 3, it is only found in 40% of experiments.

Finally, if we look at heatmap of percentage of retrieved causal relationships for dataset 8, which is shown in Figure 8, we can see both problems with TCDF interpretability. First, for time series with index 2 and 3, the interpretation is that they are generated by time series with index 2 and 3, which is wrong. Either the first or last time series generate these time series. Second, causality is missing for the last time series in 60% of experiments.

When we look at temporal causality, given by TCDF, we see that it is wrong in almost all experiments and datasets. There are usually multiple temporal casualties in our datasets, but TCDF usually gives only one, and that one is almost always not correct.

#### 4.2.3. IMV-LSTM

**Figure 9:**
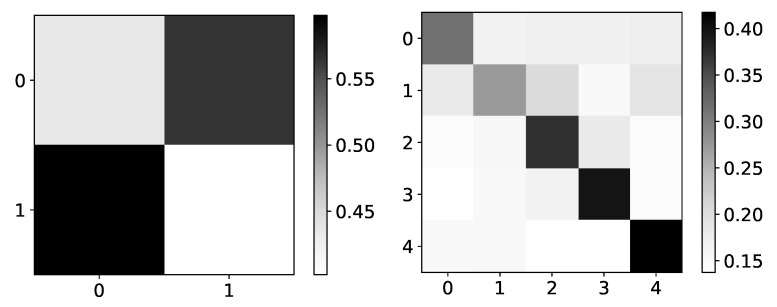
The mean values of β coefficients from IMV-LSTM model for dataset 4 and dataset 2. The y-axis corresponds to the target series index, and the x-axis corresponds to the index of series whose impact on selected target series we plot. left) In the left panel, we plot mean β coefficients of IMV-LSTM model for dataset 4. We can see that IMV-LSTM model gives correct interpretability for this dataset, but with low confidence. In the right panel, we plot mean β coefficients of IMV-LSTM for dataset 2. Again, IMV-LSTM model gives correct interpretability, but with high confidence on this dataset.

The only model that shows a reasonable interpretation of most datasets is IMV-LSTM. In Figure 9 we plot mean values of β coefficients for dataset 4 (left) and dataset 2 (right). If we look at mean β values for dataset 4, we can see that IMV-LSTM gives correct interpretability. However, confidence is still relatively low.

A comparable result can be seen on dataset 2, shown on the right panel in Figure 9, correct interpretability, but with high confidence. For simple datasets, IMV-LSTM gives correct interpretability for causality between different time series.

**Figure 10:**
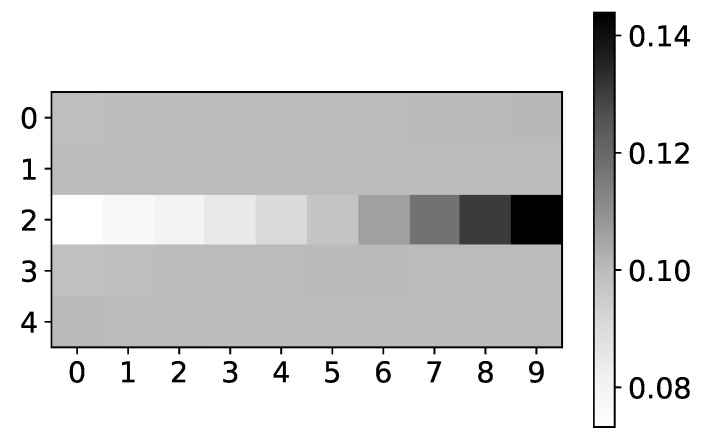
The mean values of α coefficients of IMV-LSTM model for dataset 2. The y-axis corresponds to the target series’s index, and the x-axis corresponds to the time lag whose impact on the selected target series we plot. We can see that IMV-LSTM is also biased towards the end of the window. However, it is crucial to notice that for time series which do not impact target series, α coefficients are uniform.

If we look at mean α coefficients of IMV-LSTM for dataset 2, time-series with index 2, shown in Figure 10, we can see that they are biased towards the end of the window. Again, bias towards the beginning of the window or end of the window is seen across all datasets. LSTM layer in the model is the probable cause. It is also important to note that α values usually have dynamic (differ from a uniform distribution) only in time series which have high β values.

**Figure 11:**
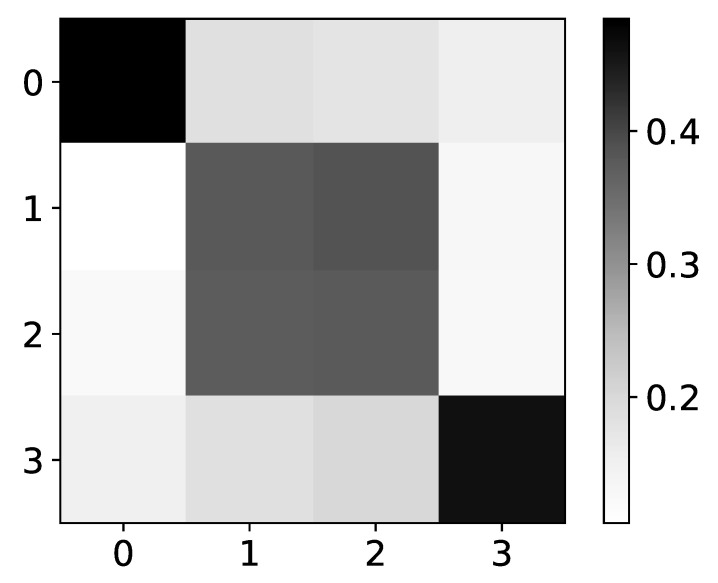
The mean values of β coefficients from IMV-LSTM model for dataset 8. The y axis corresponds to the index of target series and x axis corresponds to the index of series whose impact on selected target series we plot. We can see that IMV-LSTM gives correct interpretability for time series with index 0 and 3, whose behaviour is autoregressive. For time series with index 1 and 2, this model gives wrong interpretability.

However, for more complex datasets, IMV-LSTM model fails to give correct interpretability. In Figure 11 we show mean β values for dataset 8. For the first and last time series, we have correct interpretability.

For the second and third time series (index 1 and 2), interpretability is wrong. Different rules are used to generate these time series, based on the value of time series 0. This dataset is possibly too complex for model to learn correct interpretability, so it defaults to a rather autoregressive causality. This behavior is seen with TCDF also.

#### 4.2.4. DA-RNN

Attention coefficients given by DA-RNN cannot be interpreted without additional transformations (this is true for both input attention, which models crosscorrelation, and for temporal attention). Aggregating input attention gives us a value that corresponds to β coefficients. Note that the DA-RNN model does not have interpretability connected to autocorrelations. It only models crosscorrelations using attention coefficients. Aggregating temporal attention gives us values that correspond to α coefficients. However, these α coefficients can not be mapped to the input time series. They can only be mapped to encoder outputs. DA-RNN uses LSTM as an encoder layer, making it impossible to map temporal attention to specific input time series, producing aggregated temporal interpretability.

**Figure 12:**
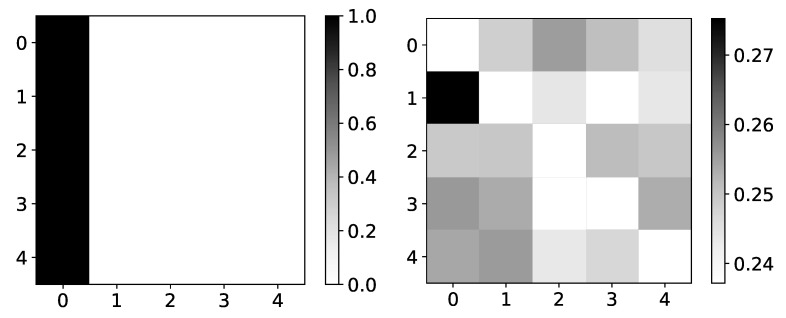
Ground truth (left panel) and aggregated β values (right panel) from the DA-RNN model for dataset 5. The y-axis corresponds to the target series index, and the x-axis corresponds to the index of series whose impact on selected target series we plot. This dataset consists of N = 5 time series that are generated from time series with index 0. This is the reason we expect high values in the first column. β coefficients of DA-RNN differ from what we expect. Furthermore, the distribution of these coefficients is highly uniform.

In the left panel in Figure 12, we plot ground truth for dataset 5. For this dataset, one would expect high values in the first columns, as time series with index 0 is the one that generates all other time series. In the right panel, in Figure 12, we plot β coefficients for DA-RNN for dataset 5. Values are missing on the main diagonal as DA-RNN does not model autocorrelation with attention. As we can see, DA-RNN does not produce expected values. Values are highly uniform, with a slight bias towards the first time series. We cannot evaluate interpretability on datasets 2 and 3 because the time series in these datasets are autoregressive. Furthermore, it is not meaningful to analyze interpretability on dataset 4, as DA-RNN gives only one attention coefficient (attention coefficient need to sum to 1).

**Figure 13:**
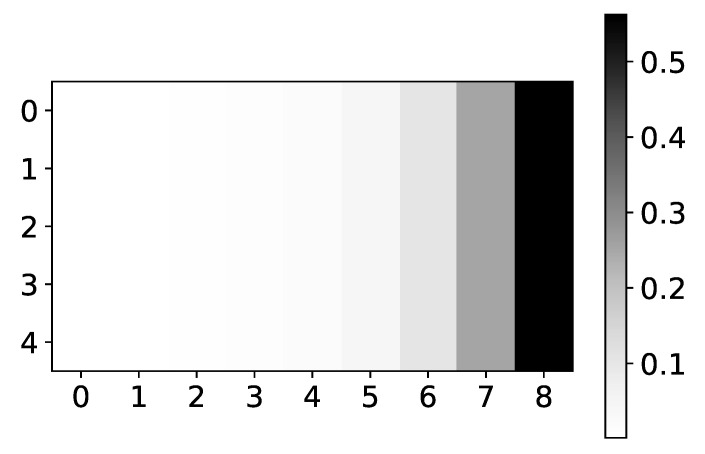
The mean values of α coefficients of the DA-RNN model for dataset 7 for time series with index 0. The y-axis corresponds to the index of feature in Encoder output, and the x-axis corresponds to the time lag whose impact on selected target series we plot. We can see that DA-RNN is biased towards the end of the window. The same behavior is seen with seq2graph and IMV-LSTM.

In Figure 13, we plot α coefficients for dataset 7 for time series with index 0. Here, the y-axis does not correspond to the input time series index but to the index of feature in Encoder output. DA-RNN has the same problem as IMV-LSTM and seq2graph, i.e., models that use sequential layers. They are either biased towards the beginning or end of the window. We suspect this behavior originates from these sequential layers.

### 4.3. Sensitivity Analysis

In Section 4.1, we analyzed the performance of the models, and here we are only interested in the effect of hyperparameters (that generate time series) on models performance, i.e., we will look at average error divided by maximum model error.

#### 4.3.1. Dependency on Noise Frequency

**Figure 14:**
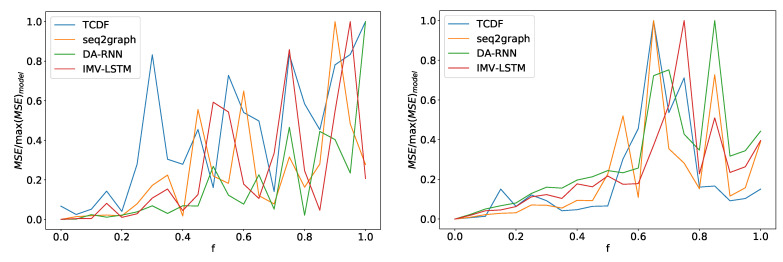
In the left panel, we can see how the maximum error percentage for specific model changes with *f* for dataset 2. We observe a change in behavior at around f=0.4. In the right panel, we can see results for dataset 7. Here we see a change in behavior around f=0.6. All models show similar behavior with a slight deviation from the TCDF model on dataset 2.

In the left panel in Figure 14 we can see average error divided by maximum model error for four models depending on noise frequency *f* for dataset 2. For all models, error increases as the control parameter increases. We can see somewhat of phase transition at f=0.4. After this point, performance for all models oscillates heavily with the noise frequency parameter. All models show a similar pattern, and there is a slight deviation for the TCDF model. Its performance deteriorates a bit quicker than other models. At f=0.3, it has 80% of maximum model error, while IMV-LSTM achieves 80% maximum loss at almost a f=0.9. Similar transition behavior is also seen on dataset 7, where we have a phase transition around f=0.6, as shown in the right panel in Figure 14. On this dataset, TCDF behaves in the same way as other models.

#### 4.3.2. Dependency on Noise Amplitude

**Figure 15:**
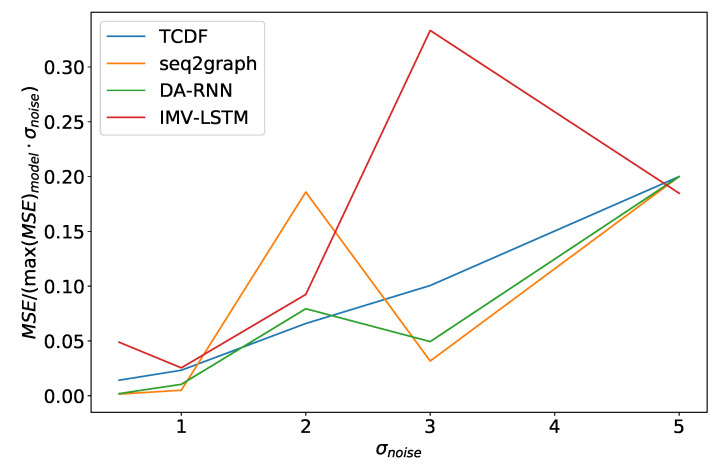
Maximum error percentage divided by $\sigma_{noise}^{2}$ vs. $\sigma_{noise}^{2}$ for dataset 2. We see that models have an almost linear dependency on $\sigma_{noise}^{2}$, with TCDF achieving almost perfect linearity.

Similar effects can also be seen in experiments where we vary the noise amount $\sigma_{noise}^{2}$. In Figure 15, we can see the average model error in percentage of maximum model error, divided by $\sigma_{noise}^{2}$, for the dataset 2. It is important to normalize the percentage error by noise amplitude in the experiment because of how we generated data. In most cases, $\sigma_{noise}^{2}$ is the parameter that defines the order of magnitude for time series.As we can see, in Figure 15 models have an almost linear dependence on $\sigma_{noise}^{2}$, with the TCDF model having nearly perfect linear dependence. The same effect is seen in dataset 7. The only difference is that TCDF has a curve more similar to other models. Linear dependency between normalized error and $\sigma_{noise}^{2}$ is interesting because it suggests that noise amplitude does not just increase error in points where we add noise but makes it harder for the model to learn correct behavior overall.

#### 4.3.3. Dependency on Number of Time Series

**Figure 16:**
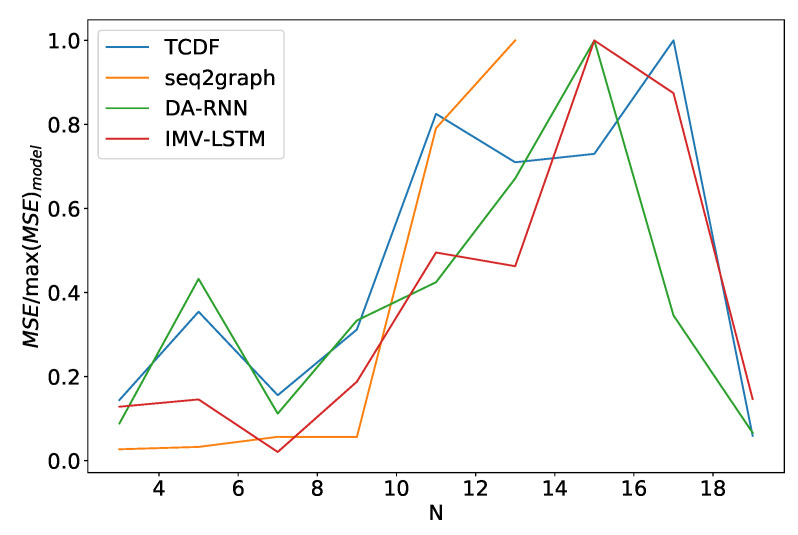
Percentage of maximum model error vs. number of time series *N* for dataset 2. We can see a significant increase in error percentage at N=10. Notice that seq2graph only goes to N=13. For higher *N*, seq2graph model had memory problems.

Finally, we look at how these models perform when we vary the number of time series *N* they model. We see somewhat similar performance as in the noise frequency experiment. In Figure 16 we plot the average model error percentage for the dataset 2 for different values of *N*. The curve for seq2graph model only goes to N=13 time series. Reason for this is that seq2graph model had memory problems when we surpassed N=13 time series. All models have similar behavior, and we see an abrupt change at N=10.

**Figure 17:**
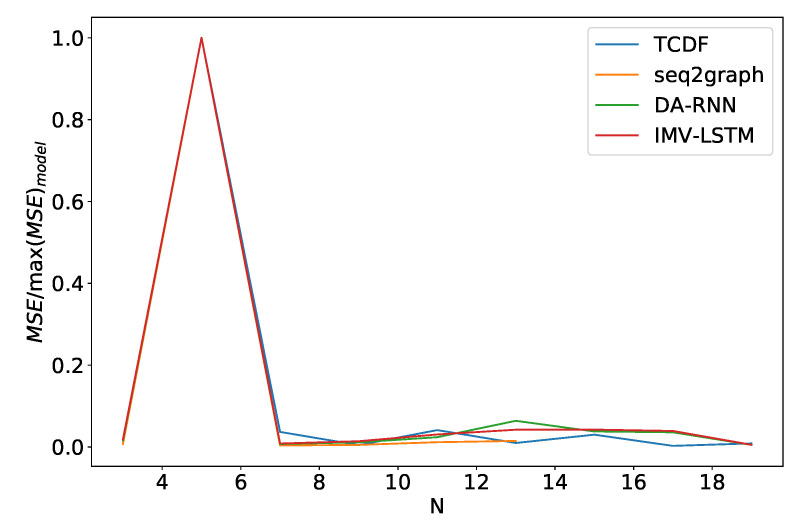
Percentage of maximum model error vs. number of time series *N* for dataset 5. For this dataset percentage of the maximal model error is almost independent of N, and this behavior is almost identical for all models.

The performance of models on dataset 5 is different from what we saw with the dataset 2, as shown in Figure 17. The number of time series has almost no effect on models, besides outlier at N=5. Furthermore, all models have almost identical behavior. We suspect that this effect occurs because time series are entirely driven by time series with an index 0. Models only need to understand that time series 0 is a generator and find the correct mapping. On dataset 2, which is *N* independent autoregressive time series, the model needs to understand that every time series is autoregressive and then find the correct mapping.

### 4.4. Simulated Data from Statistical and Mechanistic Models

Since IMV-LSTM is the only model (from the ones that we investigated) that shows correct interpretation for most datasets, we will further investigate IMV-LSTM interpretability on time series generated from the Ising model and Logistic map inspired model.

#### 4.4.1. Ising Model

**Figure 18:**
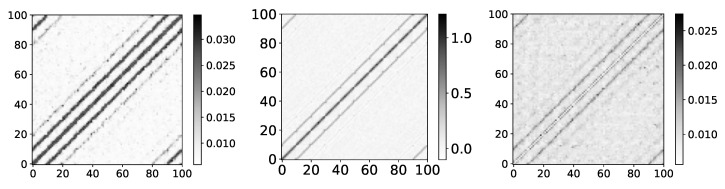
The y axis corresponds to the index of target series and x axis corresponds to the index of series whose impact on selected target series we plot. On left image we plot mean β values for dataset 9 for T=2.75. Model shows that first neighbours have highest impact on targeted spin with diminishing impact for higher order neighbours. Spin correlation given by RMB approach. We can see high similarity between these values and values given by IMV-LSTM. Same graph as left, but with T = 2. At temperatures lower than critical, spins become frozen and we have long-range correlations. This long-range correlation is what makes this graph more blurry.

To analyze the performance of IMV-LSTM at Ising dataset, we ran ten experiments. If we look at the left panel in Figure 18, showing mean β coefficients for IMV-LSTM, we can see that model discovered correct interactions. We can confirm this by comparing results with results of [31]. In this paper, the authors used Restricted Boltzmann Machines (RBM) to extract correlations between spins. They look at the matrix $WW^{T}$, where *W* is the weight matrix that maps the visible layer to the hidden layer. Authors argue that the product of weight matrices $WW^{T}$ must reflect correlations between spin variables of the input configurations. Their result for $WW^{T}$ is practically identical to the result shown in Figure 18.

It is necessary to stress that IMV-LSTM for Ising was trained differently from RBM. RBM has two fully connected layers, one visible and one hidden, and it is trained with standard RBM loss. IMV-LSTM is trained to predict each spin’s direction in the next time step, with MSE loss. Furthermore, the figure generated by IMV-LSTM is somewhat more blur than the one produced by RMB. We attribute this blurriness to LSTM layers and attention mechanism, which are known to dilute importance (the opposite of a fully connected layer that is not considered a spatial part of time series). If we compare β coefficients on temperature before and after phase transition, we can see that the figure becomes even more blurred on a lower temperature (shown in the right panel in Figure 18). This is expected behavior because spins become frozen at a lower temperature, i.e., they do not change state over many time steps, causing long-range correlations.

**Figure 19:**
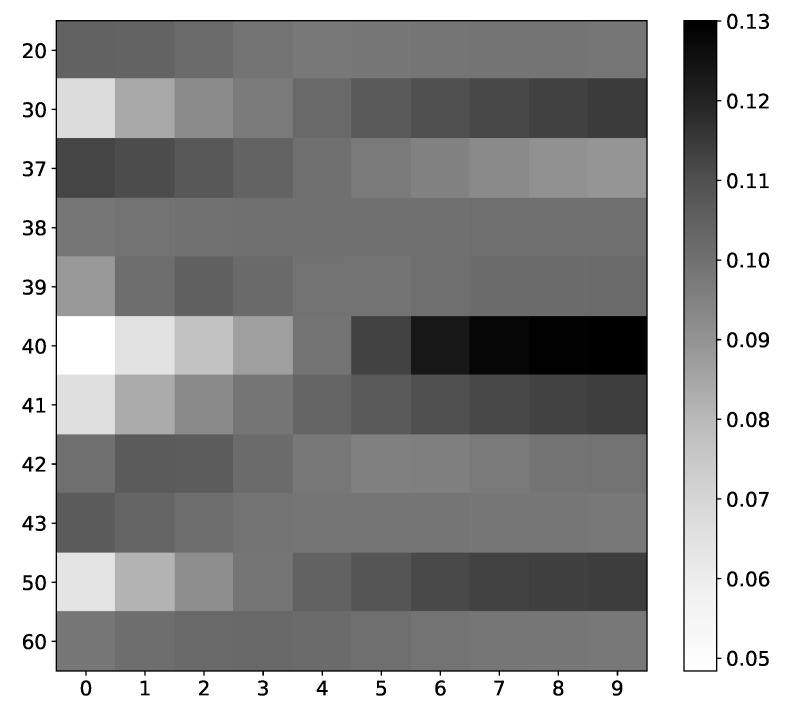
IMV-LSTM mean α coefficients for spin #40. The x-axis corresponds to the time lag, and the y-axis corresponds to the index of spin. We only show interaction with selected spins, as there are 100 of them. As we can see, spins that interact with our selected spin have the most diverse values. Spins that do not interact with spin #40, for instance, spin #20 or spin #37, have almost uniform values across all time stamps.

For most spin values in dataset 9, mean α values (Figure 19) are near uniform with a slight increase or decrease towards the edge of the window. However, spins that impact the next state of spin that we analyze have an importance coefficient with a higher range, i.e., the model prefers specific time steps for these spins. Here, preferability is correctly assessed; the model gives the highest importance to the last time step, which is the lag we used to generate the dataset. The highest importance score is seen in the spin that we analyze.

This is also one of the more interesting properties of IMV-LSTM. Even from α coefficients, one can see which time series impact output. This helps us understand how the model makes a prediction and helps us understand how the model is trained, which is a step towards more explainable neural networks.

#### 4.4.2. Logistic Map Inspred Model

**Figure 20:**
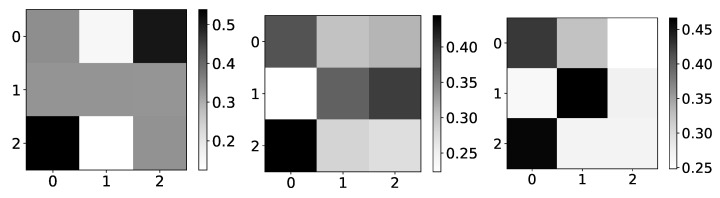
The mean values of β coefficients from IMV-LSTM model for dataset 10 for 3 different values of *r*: 1.5, 3.55 and 3.56996. The y-axis corresponds to the index of target series and x-axis corresponds to the index of series whose impact on selected target series we plot. left) In the left panel we plot mean values of β coefficients for r=1.5. As we can see, the interpretability is completely wrong, but in this regime, logistic map converges to single value. In the middle panel we plot mean values of β coefficients for r=3.55. As we can see, the interpretability is much closer to expected behaviour. In this regime, logistic map oscillates between several different values, so it is beneficial to model to learn correct interpretability. In the right panel we plot mean values of β coefficients for r=3.56996. As we can see, the interpretability is almost correct with high confidence.

Rather interesting results can be seen on Logistic map data, i.e., dataset 10. The importance given by the model is becoming more and more correct when we approach chaotic behavior (Figure 20). One of the reasons for this could be that, while we are far from chaotic behavior, that is, when data oscillate between two values, there is no reason for the model to learn correct interpretability. The model can learn anything and still make correct predictions. This would be a somewhat underutilization of model capabilities. Furthermore, when the model oscillates between a few values, there is no fundamental reason that learning to copy only this value is wrong interpretability. Similar phenomena are seen on the Ising dataset, where importance coefficients are a lot more dispersed at lower temperatures caused by frozen spins. Frozen spins mean that spins stay in the same state for most of the time and only change state on rare occasions. The model learns to map the last state and does not bother to learn true causality.

Once we enter chaotic behavior, we can see that we have correct interpretability for the logistic map time series, i.e., time series with indexes 1 and 2. However, for the third time series, which is just the average of the first 2, we have the wrong interpretation. It is essential to state that IMV-LSTM has great performance on this dataset, qualitative wise, i.e., mean squared error is extremely small. For the low *r* parameter, it is practically zero. This means that IMV-LSTM can create correct predictions without fully transforming its weights into something interpretable to humans.

## 5. Conclusions and Outlook

This paper analyzed four different neural network architectures with inherent interpretability (or causal neural networks) for multi-time series analysis: seq2graph, IMV-LSTM, TCDF, and DA-RNN. An analysis of the confidence and correctness of networks’ interpretability, not just prediction performance, was the central point of this paper. We utilize synthetic datasets with increasing complexity levels of interaction between multiple time series as a benchmark for validating models’ interpretability.

For instance, dataset 2, a set of N independent autoregressive time series, showed us that seq2graph could not correctly interpret the data generating process. Dataset 4 is the first dataset that incorporated interaction between different time series. It showed us that the TCDF model could not interpret this interaction between time series even though it can model auto-causality easily. Dataset 8 showed us that IMV-LSTM while giving correct interpretability for all other datasets, breaks when we incorporate switch-like behavior to our data. TCDF and seq2graph did not produce correct interpretability for most datasets (TCDF only gave correct interpretability for dataset 2). Also, seq2graph had low confidence in given interpretability. The model that produced correct interpretability for most of the datasets was IMV-LSTM. Interestingly, the interpretability given by TCDF and IMV-LSTM on dataset 8 is almost identical. When we looked at temporal interpretability, both seq2graph and IMV-LSTM, which are RNN based neural networks, were biased to either beginning or the end of the window. We assume this is a consequence of the usage of RNN layers.

Evaluated attention-based models have satisfying MSE performance on these datasets, even though they do not give correct interpretability. Models can learn to predict without understanding the underlying data generating process. Prediction performance, measured by MSE, ranked IMV-LSTM as the best performing model, followed closely by the DA-RNN and seq2graph model. The worst performing model was TCDF. Stability of performance, measured by the variance of MSE, showed that TCDF had the largest variance, while IMV-LSTM had the lowest, which positioned the IMV-LSTM model as both the best-performing one and the most stable one.

Datasets 9 and 10 (statistical and mechanistic model, respectfully) showed us interesting behavior. The increasing complexity of the underlying process (increasing probability of spin switch) for dataset 9 or going from oscillating between two values in time series to a more chaotic one for dataset 10 shows an increase in interpretability’s correctness. Interpretability given by IMV-LSTM on first-order 2D squared Ising lattice was correct, which we can confirm by comparing it with RBM’s interpretability. On the logistic map dataset, we saw interesting behavior of interpretability coefficients. As we approach the more chaotic regime of the logistic map, interpretability is becoming more and more correct. This brings up interesting phenomena of interpretability. If a dataset is too easy to learn, there is no point for a model to produce correct interpretability. The model can learn how to make a correct prediction without learning the mechanics which generated the data. This is also seen on the Ising dataset with lower temperatures, where spins stay in the same state for a longer time. For more complex problems, it is beneficial for a neural network to learn the underlying process.

Experimental settings based on artificially created transparent benchmark datasets provided us with insightful weaknesses in current state-of-the-art, attention-based architectures’ performance and behavior. Motivated by these insights, future work will focus on designing and experimenting with novel neural architectures that can provide more stable and faithful interpretability. However, the limitation of our study is that our experimental settings are mainly focused on multivariate time series single output forecasting tasks and specific well-known generative processes applicable to some real-world domains. We plan to extend our experimental setting with other multivariate prediction tasks, including multi-input/multi-output classification and forecasting settings from various domains that include real-world datasets.

## Figures and Tables

**Table 1:** Models used for benchmarking, with each successive dataset model’s complexity is increased: constant time series (dataset 1) to autoregressive (dataset 2) and nonlinear autoregressive (dataset 3) with no interaction between time series, two interdependent time series without autoregression (dataset 4), first series is autoregressive (dataset 5) and nonlinear autoregressive (dataset 6) time series with all other time series calculated from first, custom vector autoregression model (dataset 7), switching time series (dataset 8). Additionally, we created two datasets from statistical and mechanistic models: The logistic map inspired model (dataset 9) and the Ising model on the first-order 2D square lattice (dataset 10).

Name	Formula	Parameters
Dataset 1	$X_{n,t}=C_{n}+\epsilon_{t}$	$C_{n}=N\left(0,1\right)$
Dataset 2	$X_{n,t}=c_{t_{lag}}X_{n,t-t_{lag}}+\epsilon_{t}$	$c_{3}=1/2,c_{7}=1/2$
Dataset 3	$X_{n,t}=\tanh\left(c_{t_{lag}}X_{n,t-t_{lag}}+\epsilon_{t}\right)$	$c_{3}=5/7,c_{7}=1/7,c_{9}=1/7$
Dataset 4	$X_{1-n,t}=c_{t_{lag}}X_{n,t-t_{lag}}+\epsilon_{t}$	$c_{2}=2/5,c_{5}=1/5,c_{9}=2/5$
Dataset 5	$X_{n,t}=c_{n,t_{lag}}X_{0,t-t_{lag}}+\epsilon_{t}$	$c_{0,3}=1/2,c_{0,4}=1/2$ $c_{1,9}=1$ $c_{2,2}=1/2,c_{2,7}=1/2$ $c_{3,3}=1/10,c_{3,4}=1/10,c_{3,8}=4/5$ $c_{4,2}=1/3,c_{4,5}=2/9,c_{3,8}=4/9$
Dataset 6	$X_{n,t}=\tanh\left(C_{n,t_{lag}}X_{0,t-t_{lag}}+\epsilon_{t}\right)$	$c_{0,3}=1/2,c_{0,4}=1/2$ $c_{1,9}=1$ $c_{2,2}=1/2,c_{2,7}=1/2$ $c_{3,3}=1/10,c_{3,4}=1/10,c_{3,8}=4/5$ $c_{4,2}=1/3,c_{4,5}=2/9,c_{3,8}=4/9$
Dataset 7	$X_{0,t}=c_{0,1}X_{0,t-1}+c_{0,5}X_{0,t-5}+\epsilon_{t}$ $X_{1,t}=1+c_{1,2}X_{0,t-2}+\epsilon_{t}$ $X_{2,t}=c_{2,1}X_{1,t-1}+c_{2,4}X_{3,t-4}+\epsilon_{t}$ $X_{3,t}=1+c_{3,4}X_{2,t-4}+c_{3,1}X_{0,t-1}+\epsilon_{t}$ $X_{4,t}=c_{4,4}X_{4,t-4}+c_{4,1}X_{1,t-1}+\epsilon_{t}$	$c_{0,1}=1/4,c_{0,5}=3/4$ $c_{1,2}=-1$ $c_{2,1}=1,c_{2,4}=1$ $c_{3,4}=-2/7,c_{3,1}=5/7$ $c_{4,4}=12/22,c_{4,1}=10/22$
Dataset 8	if $X_{0,t-5}>1/2:$ $X_{0,t}=c_{0,1}X_{0,t-1}+c_{0,3}X_{0,t-3}+\epsilon_{t}$ $X_{1,t}=X_{0,t-5}+\epsilon_{t}$ $X_{2,t}=X_{0,t-4}+\epsilon_{t}$ $X_{3,t}=c_{3,1}X_{3,t-1}+c_{3,4}X_{3,t-4}+\epsilon_{t}$ else: $X_{0,t}=c_{0,1}X_{0,t-1}+c_{0,3}X_{0,t-3}+\epsilon_{t}$ $X_{1,t}=X_{3,t-2}+\epsilon_{t}$ $X_{2,t}=X_{3,t-4}+\epsilon_{t}$ $X_{3,t}=c_{3,1}X_{3,t-1}+c_{3,4}X_{3,t-4}+\epsilon_{t}$	$c_{0,1}=1/2,c_{0,3}=1/2$ $c_{3,1}=1/2,c_{3,4}=1/2$
Dataset 9	$H\left(\sigma \right)=-\sum_{\langle i,j\rangle }J_{i,j}\sigma_{i}\sigma_{j}-\mu \sum_{j}h_{j}\sigma_{j}$	*T* = 2, *T_c_*, 2.75
Dataset 10	$X_{0,t}=rX_{0,t-3}\left(1-X_{0,t-3}\right)$ $X_{1,t}=rX_{1,t-5}\left(1-X_{1,t-5}\right)$ $X_{2,t}=1/2X_{0,t-3}+1/2X_{1,t-5}$	r = 1.5, 2.5, 3.2, 3.55, 3.56996

**Table 2:** Model prediction performance on all datasets. The average experiment MSE for each model is reported as a score. We do not report seq2graph results on dataset 9 because the model had memory problems. Dataset 9 consists of 100 series in our experiment, and seq2graph cannot model that many time series. ES-RNN model (the winner of the M4 competition) is added for comparison and evaluated only on datasets 1–8. ES-RNN is only used in quantitative analysis since it does not provide interpretability.

Dataset	DA-RNN	IMV-LSTM	seq2graph	TCDF	ES-RNN
1	0.00293 ± 1 × 10^−5^	0.02905 ± 9 × 10^−7^	0.00303 ± 9 × 10^−5^	0.0033 ± 0.0002	0.003731 ± 1 × 10^−6^
2	0.00150 ± 6 × 10^−5^	0.0018 ± 0.0002	0.011 ± 0.007	0.02 ± 0.01	0.001496 ± 9 × 10^−6^
3	0.00013 ± 1 × 10^−5^	0.000125 ± 6 × 10^−6^	0.00001 ± 2 × 10^−5^	0.0006 ± 0.0004	0.000137 ± 1 × 10^−6^
4	0.000244 ± 2 × 10^−6^	0.000238 ± 5 × 10^−6^	0.00032 ± 6 × 10^−6^	0.002 ± 0.002	0.00045 ± 1 × 10^−4^
5	0.00229 ± 7 × 10^−5^	0.00138 ± 3 × 10^−5^	0.0020 ± 0.0001	0.005 ± 0.003	0.017833 ± 1 × 10^−6^
6	0.00210 ± 1 × 10^−5^	0.00143 ± 6 × 10^−5^	0.00213 ± 0.0001	0.005 ± 0.002	0.018541 ± 1 × 10^−6^
7	0.009 ± 0.001	0.0051 ± 0.0006	0.008 ± 0.001	0.021 ± 0.007	0.0120 ± 0.0002
8	0.286 ± 0.006	0.258 ± 0.002	0.18 ± 0.05	0.3 ± 0.1	0.250 ± 0.001
9	0.3353 ± 0.0005	0.2688 ± 0.0001	-	0.348 ± 0.007	-
10	0.002 ± 0.001	(9 ± 1) × 10^−5^	0.006 ± 0.008	0.03 ± 0.01	-

## Data Availability

Code used for the generation of all presented datasets is available on the GitHub repository (https://github.com/hc-xai/mts-interpretability-benchmark).
